# Notes and News

**Published:** 1889-02-23

**Authors:** 


### NOTES AND NEWS.

COLLECTED AND COMMUNICATED.

"VOYAGES IN VACATION" is unavoidably postponed until next week.

THE annual meeting of Richmond Hospital subscribers takes place to-day. The financial statement shows: Income, £5,216 1s. 5d. (including £2,249 1s. 7d. legacies, duly invested); balance in hand, £92 14s., as compared with £163 17s. 5d. last year.

MR. A. B. BOLTON and several of the nurses of St. George's Hospital gave an entertainment on Tuesday, 12th, at St. Barnabas Boys' School, Pimlico, on behalf of the Church of England Temperance Society. An agreeable programme was carried through very successfully, and received with much applause.

LADY ELLIS held a drawing-room meeting at Buccleuch House last week in aid of the Society for the Prevention of Cruelty to Children. The Baroness Burdett-Coutts made an excellent speech, and the Duchess of Teck made many practical inquiries about the working of the society, which showed a true interest in the poor children whom the society seeks to benefit.

AT the annual general court of the City of London Lying-in Hospital, held on February 15th, the report stated that during the past twelve months there had been 410 in-patients and 1,354 out-patients. This was the largest number that had been benefited in any year since the formation of the charity in the year 1770. After all expenses had been defrayed, a sum of £454 remained to be carried forward.

THE operation of inserting an animal's eye into the human eye-socket is reported to have been sometimes carried out with success. In one case, however, the rabbit's eye, which was to have been transplanted, was accidentally injured, and the eye of the domestic cat (which happened to be in the room at the time) was used instead. The patient recovered, and the doctors regarded it as a very good case; but still the patient wasn't satisfied. It was all right in the daytime he avered, but at night he couldn't get a wink of sleep, that wretched eye always being on the look-out for rats!

THERE are upwards of a thousand families identified with the King Edward Ragged Schools and Mission, Spitalfields, and many of the poor children and aged people are suffering very much from want of boots, firing, etc. Many benevolent people who used to help cases of special distress have passed away, and it is necessary to apply to others for assistance, and, as the committee have no funds at their disposal for these objects, an earnest appeal is now being made for contributions to assist in mitigating the sufferings of these poor children and grown-up people. The honorary superintendent is [illegible]r. Charles Montague, to whom contributions may be sen[illegible] [illegible]he late Lord Shaftesbury was president of the miss[illegible]til his death. The present president is the Earl of Aber[illegible]

A CONCERT was given last Saturday in aid of a new wing for the Morley House Seaside Convalescent Home. The proposed wing is to be called the Caxton wing, and is be built and supported chiefly by printers. The Morley House Home is noted us being an institution for workmen, managed and kept by workmen, with the result that irksome restrictions which exist at other homes are here conspicuous by their absence.

AT a meeting of the committee of the New Hospital for Women, held at 222, Marylebone Road, when the Hon. Maude Stanley, Mrs. Garrett-Anderson, M.D., Lady Jackson, Mrs. J. Hollond, Mrs. Westlake, and others were present, an address was presented to the Rev. J. Llewellyn Davies, expressing deep regret at the prospect of losing his services as their chairman, owing to his removal from London; also their gratitude for much help and kindness, and most earnest wishes for his happiness and welfare in his new sphere.

THE annual meeting of the East London Nursing Society is to be held at the Mansion House to-day (Feb. 22nd). The necessity of starting a fourth matron to look after the increasing number of districts nursed by the society makes the appeal now issued for further subscriptions most urgent. A matron ought always to visit every district once a week, and it takes a day to do a thorough round; so that a matron with more than seven districts is obviously over-worked. During last year the nurses of the society attended to over 3,000 patients.

FOR the last three years ambulance classes have been held at Bolton Infirmary, and at a grand function held lately Mr. and Mrs. W. Slater distributed numerous medallions and certificates obtained by successful pupils. Mr. T. Cooper then rose and said he had been requested by the members of the male classes to present to Dr. Yates in their name a beautiful time-piece, as a token of their appreciation of his labours. Mr. Bradbury then presented Dr. Yates and Dr. Kingsford each with a dressing-case from the lady members of the class. Then somebody presented somebody else with a pipe and a walking-stick, and after much mutual congratuation the bearers of medals and presentations dispersed.

MR. BRAXTON HICKS, the Surrey Coroner, has reopened the discussion on infantile life assurance, a subject which is just now vexing the members of the Societiy for the Prevention of Cruelty to Children. Mr. Hicks does not propose total abolition of the system, but he would surround it with a number of restrictions, the most sensible of which is, that the societies should insure a funeral and not the payment of a sum of money. Since one child in five dies before attaining the age of two, it is not to be wondered at that poor parents insure against the risk; it would scarcely be just to refuse to let them do so, but in so far as the right has been horribly abused (as physicians know only too well), it is to be hoped that the suggestions of Mr. Braxton Hicks will be attended to, and in time enforced.

NEWCASTLE ROYAL INFIRMARY reports that last year's expenditure reached £11,801, and the income amounted to £11,005: this leaves a deficit of over £700. The in-patients last year numbered 3,175, each patient staying on an average 27 days, at a cost of £3 12s. 2½d. The operation of ovariotomy was performed nine times, of which eight were successful. The excission of the patella was successfully performed once, also an operation by which the kidney was stitched to the loin. There have been many changes in the medical staff of the Newcastle Infirmary lately. Dr. Luke Armstrong is greatly missed; so have been Dr. Cave and Dr. Gibbard, both convalescent now from typhoid fever. Mr. S. J. Alden is the new house-surgeon in the place of Mr. Wilkinson.

THE following vacancies are announced this week, the amount of salary in each case being given after the name of the institution :—

**Resident Medical Officers.**

West Herts Infirmary. House Surgeon and Dispenser.—£100, board, etc.
Tunbridge Wells General Hospital. House Surgeon and Secretary.—£100, board, etc.
Birmingham General Hospital. Medical Officer.—£130, board, etc.
Royal London Ophthalmic Hospital. Assistant House Surgeon. —£50, board, etc.
South Devon Hospital, Plymouth. Assistant House Surgeon.—Board, etc.
Birmingham Homœopathic Hospital. House Surgeon.—£80, board, etc.

**Non-resident Medical Officers.**

Chelsea Dispensary, Sloane Square. Surgeon.
Sheerness Medical Society. Surgeon.—£300.
London Skin Hospital. Medical Officer.
City of Manchester. Medical Officer of Health.—£850.

A UNIVERSAL Cookery and Food Exhibition will be held at the Knightsbridge Riding School from April 30th to May 10th, in aid of the Great Northern Central and other hospitals. The exhibits will include artistic cookery of all sorts, and confectionery in its plainest and most fanciful stages. Uncooked provisions, preserved provisions, and table decorations will also form special features. The exhibition ought to be highly interesting to all who study health questions.

LORD BALFOUR OF BURLEIGH has been elected president of the London Fever Hospital. During last year 603 cases were treated in this hospital the rate of mortality being 3·1 per cent. The great point with regard to this hospital is not so much the cases it cures as the cases it prevents. One fever patient treated here, if treated at home, would very probably give the disease to several other persons. The charge for the necessary six weeks' treatment is three guineas; and a balance of £576 has been carried on to the hospital's credit.

ONCE more we have news of Father Damien, and this time it tells of the arrival of Mr. Clifford at Molokai with all the presents sent to the lepers by our sympathic English folk. Mr. Burne Jones sent a picture which now hangs in Father Damien's room; Lady Mount-Temple sent an engraving of "The Good Shepherd;" a hand organ, a magic lantern, and other trifles were sent by other friends. All these evidences of English appreciation are very dear to Father Damien. Mr. Clifford also took with him large quantities of gurjun oil, which is the latest reported cure for leprosy. So far it seems at least to have given relief, and few of us expect more than that from its powers. There are over 1,000 lepers at the settlement, and they are of many nationalities and faiths, but father Damien is the slave of them all. No job is too menial for him—building, carpentry, tending the sick and washing the dead, are all part of his daily life.

THE governors of the Charing Cross Hospital held their annual court at the hospital last week. Mr. R. B. Martin (the treasurer) presided, and moved the adoption of the report, which stated that during the year 1888 no fewer than 1,870 in-patients had been admitted, a number larger by nearly two hundred than any hitherto recorded in the history of the hospital. In the out-patient department 9,617, including 209 midwifery cases, and in the casualty-rooms 9,735 patients had been treated; making a grand total of 21,222. The heavy overdraft at the bankers on December 31st, 1887, amounting to £5,154, had been reduced to £466, and it was stated that the deficit would entirely have disappeared but for unforeseen delays in the receipt of sundry payments. To the improved financial position of the hospital the very successful festival dinner held at the Hotel Metropole, under the presidency of Lord Derby, had largely contributed.

A DELIGHTFUL fête in aid of the Grosvenor Hospital for Women and Children has been planned. A new idea has been hit upon of discarding the usual stage canvas and paint effects, and of building a charming country scene with the material which Nature herself supplies. For example, there will be veritable corn ricks, moss-covered cottages, a practical windmill, a vinery, a rose-bower, an old caravan filled with toys for the children, etc., on the garden terrace, tableaux of the famous Watteau pictures will be represented, and there will be fruit and flower displays and competitions. The Princess of Wales, Princess Beatrice, and many distinguished ladies have lent their patronage to the fête, which will take place on May 29th, 30th, and 31st, at the Royal Albert Hall. It is hoped to raise a large part of the £15,000 sorely needed to rebuild the hospital.

As soon as the first breath of spring is on us, the question of boarding out will come to the fore once more. It is a pity that the society in Buckingham Street is not going to publish its report till Easter, for the earlier this matter is put before the public the better. How much good a London child gets out of a fortnight in the country it is impossible to estimate; but this we may say, that the benefit is to soul as well as body. The simple country ways, the beauty of earth and sky, and the bounty of Nature, must all impress on the child's mind such thoughts and dreams as would never have arisen in a London slum. To be even for two weeks free from the wear and worry, the noise and strife of town life, and to roam in the still country lanes and breathe the uncontaminated air of heaven, must give every child a knowledge of beauty and purity to glorify its otherwise unglorious life. Individual effort has done much to secure a period of rest and change to different divisions of the working world lately, and amongst these efforts one of the best was undertaken last summer by a medical student. He carried off thirty East-end lads to Anglesey, and camped out with them on the beach. They fed chiefly on oatmeal, bacon, and tinned food, for fresh meat was dear and troublesome to cook. They contracted for milk and bread and eggs, and they fared plainly, and spent the days in long excursions, in learning to swim, and in healthy amusements generally. The cost, including railway fare, was under £2 a-head. Surely never was £60 laid out to greater advantage.

M. WADDINGTON, the French Minister, presided on February 9th at the twenty-first annual dinner in aid of the funds of the French Hospital and Dispensary. The large company included the Italian Chargé d'Affaires, M. Tusserand, Vicomte de St. Genys, Baron Da Costa Ricci, Mr. Sheriff Newton, and many other gentlemen. Since its opening in December, 1867, the institution has afforded relief to 5,165 in-patients and 122,105 out-patients, divided into no fewer than thirty nationalities, for, although French in name, it is cosmopolitan in character. The total receipts in 1888 were £3,431, showing a decrease of £120. The expenditure was £2,099 for maintenance and £262 for management, a proportion showing that the institution is administered with great economy. At the conclusion of the report, M. Waddington gave the toast of "The Queen, the Prince and Princess of Wales, and the Royal Family," and, in doing so, said he especially asked his compatriots to respond to the toast, because the Queen was going across the Channel to spend a month in their country. M. Waddington also toasted the founders and benefactors of the French Hospital, and made the interesting announcement that the late M. Hauchois had left a legacy to the institution of one million francs (£40,000). The funds of the hospital were in a satisfactory state, but a great work had to be maintained, and he eloquently appealed for donations to carry on and develop the work. Other toasts followed, and during the evening the audience were entertained by the musical contributions of Cavaliere Tito Mattei (piano), Mdle. Vittoria de Bono (violin), and Mdle. Noémi Lorenzi and Signor Abramoff (vocalists). Donations to a large amount were received.

